# Place of care in the last three years of life for Medicare beneficiaries

**DOI:** 10.1186/s12877-023-04610-w

**Published:** 2024-01-25

**Authors:** Haiqun Lin, Irina B. Grafova, Anum Zafar, Soko Setoguchi, Jason Roy, Fred A. Kobylarz, Ethan A. Halm, Olga F. Jarrín

**Affiliations:** 1 School of Nursing, Rutgers The State University of New Jersey, Newark, NJ USA; 2 School of Public Health, Rutgers The State University of New Jersey, Piscataway, NJ USA; 3 Edward J. Bloustein School of Planning & Public Policy, Rutgers The State University of New Jersey, New Brunswick, NJ USA; 4 Institute for Health, Health Care Policy & Aging Research, Rutgers The State University of New Jersey, New Brunswick, NJ USA; 5 Robert Wood Johnson School of Medicine, Rutgers The State University of New Jersey, New Brunswick, NJ USA

**Keywords:** Place of care, Trajectory group, Medicare beneficiaries, Group-based trajectory

## Abstract

**Background:**

Most older adults prefer aging in place; however, patients with advanced illness often need institutional care. Understanding place of care trajectory patterns may inform patient-centered care planning and health policy decisions. The purpose of this study was to characterize place of care trajectories during the last three years of life.

**Methods:**

Linked administrative, claims, and assessment data were analyzed for a 10% random sample cohort of US Medicare beneficiaries who died in 2018, aged fifty or older, and continuously enrolled in Medicare during their last five years of life. A group-based trajectory modeling approach was used to classify beneficiaries based on the proportion of days of institutional care (hospital inpatient or skilled nursing facility) and skilled home care (home health care and home hospice) used in each quarter of the last three years of life. Associations between group membership and sociodemographic and clinical predictors were evaluated.

**Results:**

The analytic cohort included 199,828 Medicare beneficiaries. Nine place of care trajectory groups were identified, which were categorized into three clusters: home, skilled home care, and institutional care. Over half (59%) of the beneficiaries were in the home cluster, spending their last three years mostly at home, with skilled home care and institutional care use concentrated in the final quarter of life. One-quarter (27%) of beneficiaries were in the skilled home care cluster, with heavy use of skilled home health care and home hospice; the remaining 14% were in the institutional cluster, with heavy use of nursing home and inpatient care. Factors associated with both the skilled home care and institutional care clusters were female sex, Black race, a diagnosis of dementia, and Medicaid insurance. Extended use of skilled home care was more prevalent in southern states, and extended institutional care was more prevalent in midwestern states.

**Conclusions:**

This study identified distinct patterns of place of care trajectories that varied in the timing and duration of institutional and skilled home care use during the last three years of life. Clinical, socioregional, and health policy factors influenced where patients received care. Our findings can help to inform personal and societal care planning.

**Supplementary Information:**

The online version contains supplementary material available at 10.1186/s12877-023-04610-w.

## Background

Older adults may require help managing their chronic conditions, taking medications, or performing personal care activities, such as eating, bathing, and dressing. The ability of patients, caregivers, and communities to support aging in place is impacted by advanced illness and the presence of multiple chronic conditions, including Alzheimer’s disease and other dementias (ADOD) [1]. Among older adults with advanced illness, the majority are hospitalized at least once during the last six months of life [2]. Planning for care needs in the months or years ahead by patients, their families, and their health care providers may help to minimize unwanted and burdensome care transitions [3]. Where and when patients receive care in institutional versus home settings has important implications for personal and societal care planning, health care costs, and quality of life [4, 5]. However, little is known about place of care trajectories during the last years of life, and thus, there is a need for new insights and understanding [6]. Place of care decisions are impacted by patients’ clinical condition, availability of family caregiving, insurance factors, and local community and health system characteristics [7, 8]. Of particular importance from a clinical and state health policy perspective is eligibility and access to long-term support and services, including home- and community-based care and institutional care [9].

Most of the existing research on place of care focuses on care transitions, care episodes, or the intensity of use of a particular health service setting [10, 11]. For example, one project using a nationally representative sample of 3447 older adults with dementia from the Health and Retirement Study during 1999–2008 found that individuals with dementia experienced more frequent transitions between the home, nursing home, and hospitals [10]. However, latent class analysis permits the identification of how various risks co-occur and what demographic and clinical factors underlie these risks [11]. Thus, research on *trajectories* has increasingly used latent classes for stratification. The goal is to identify unobserved groups of individuals based on their longitudinal measures. This approach is often embedded in growth mixture modeling or group-based trajectory modeling (GBTM) [12, 13]. The approach has been applied to group the change patterns in late life development into distinct trajectories [14–18]. The approach has previously been used to classify the clinical trajectory of ADOD [19, 20], to identify care levels among Medicare beneficiaries [21–23] and to describe the clinical course in the treatment of depression among young adults [24].

The present study fills a gap in the literature by examining heterogeneity in place of care spanning the last three years of life in a nationally representative sample of Medicare beneficiaries who died in 2018, considering clinical, social, and geographic factors that may impact one’s place of care trajectory. This study aimed to characterize patterns (timing and duration) of institutional and skilled home care usage in each quarter in the last three years of life with the GBTM approach. This study addressed the following questions: (1) What were the place of care trajectories over the last three years of life experienced by Medicare decedents? (2) What sociodemographic, clinical and regional factors were associated with the variation in place of care trajectories? (3) Did the patterns of place of care trajectories vary by state?

## Methods

### Aim, design and setting of the study

The aim of this study was to determine the trajectories for place of care in each quarter during the last three years of life among Medicare beneficiaries and the factors associated with these trajectories.

A retrospective cohort was assembled from Medicare beneficiaries who died in 2018, and a 10% random sample of the cohort was analyzed. The data came from the Centers for Medicare and Medicaid Services (CMS) Chronic Conditions Warehouse for calendar years 2015 through 2018. Using administrative (MBSF, EDB), assessment (MDS, MDS-SB, OASIS, IRF-PAI), and claims data (MedPAR, Hospice), we constructed a beneficiary-level care trajectory file with the care setting or place of care for each day of the last three years of life [25]. We then collapsed the data into twelve quarters (91-day periods) for analysis. Self-reported race/ethnicity was used to augment administrative race data [26, 27] and diagnosis of Alzheimer’s disease and other dementias was determined using methods described in a brief technical appendix (see Additional file 3]. Our study was approved by the Institutional Review Board of Rutgers University.

### Characteristics of participants

**Fig. 1:**
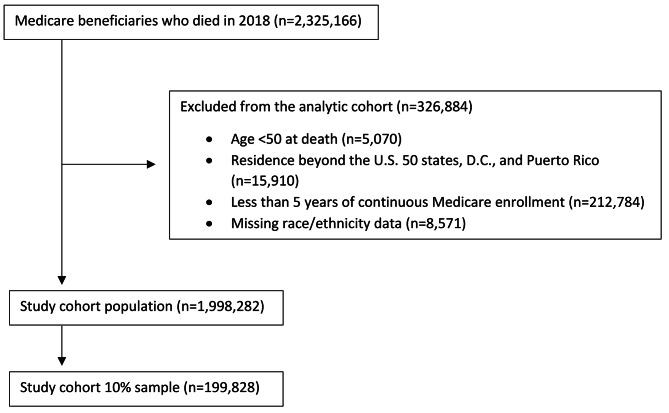
Delimitation of the analytic cohort population and sample

We selected all adult Medicare beneficiaries aged 50 and older who died in the U.S. 50 states, Washington, D.C., or Puerto Rico in 2018. The study population was limited to beneficiaries for whom race/ethnicity data were not missing (99.6%) and who were continuously enrolled in Medicare for a minimum of five years before death (90.8%). The final cohort included 1,998,282 beneficiaries, and from this population, three independent 10% random samples of 199,828 beneficiaries were selected for analysis (Fig. 1).

### Outcome variables

Place of care was summarized as the number of days in each quarter spent in each of three mutually exclusive care settings: *institutional care*, including inpatient hospitals, nursing homes, and other skilled nursing facilities; *skilled home care*, including home health care and home hospice; and *home* without skilled home care (the most frequent place of care and reference category in analyses). The care intensity during the last three years of life was calculated as the weighted average of days with skilled home care (weighted by a factor of one) and institutional/inpatient care (weighted by a factor of two). These weights were arbitrary and were assigned simply to illustrate the relative intensity of care in the last three years of life.

### Statistical analysis

To identify distinct place of care trajectory classes, we applied GBTM [12, 13]. GBTM is a statistical method used to identify latent classes that describe groups of individuals that share distinct patterns or trajectories of repeated measures. In this study, a latent class referred to a distinct pattern of bivariate repeated measures of both days of institutional care and days of skilled home care (with days of care at home without skilled home care as the reference category). The shape of trajectories was modeled using the cubic polynomial of time before death. Each beneficiary was then assigned to the trajectory class for which they had the highest posterior probability. SAS Proc Traj was used to perform GBTM [12, 28]. The data analysis was completed using SAS software, Version 9.4, SAS Institute Inc., Cary, NC, USA.

Since the number of classes was unknown in advance, we performed GBTM with two to ten trajectory classes. We selected the model with the best fit based on Bayesian information criterion (BIC) values [13] and a minimum prevalence of 2% for each class. Models with smaller BIC values indicated a better fit [29].

### Validation analysis

Three random samples of 10% Medicare beneficiaries who died in 2018 were analyzed to verify the reproducibility of the trajectory classes.

### Factors associated with place of care trajectories

The beneficiaries’ characteristics of interest were age at death, sex, race/ethnicity, insurance type, neighborhood profile [30, 31], state of residence, and chronic conditions (the list of which is given in Table 1), including dementia (see Table 1). The neighborhood profile was defined using the U.S. Department of Agriculture’s Rural Urban Continuum Codes (RUCC) and the 2018 Area Deprivation Index 3.0 (ADI 3.0) [30], a composite index of 17 socioeconomic indicators from the 2014–2018 U.S. Census American Community Survey, linked to beneficiaries’ nine-digit zip code. Consistent with the literature, neighborhoods were classified as disadvantaged if they were at or above 85th percentile (national ranking) of the ADI 3.0 [30, 31]. Areas with high or low area deprivation were further subdivided using the RUCC codes for metropolitan and non-metropolitan or rural areas: (a) urban-advantaged, (b) urban-disadvantaged, (c) rural-advantaged, (d) rural-disadvantaged [31]. The relationship between beneficiaries’ place of care trajectory class and each of their clinical and sociodemographic characteristics was assessed with cross-tabulations and multinomial regression analysis in which class membership was regressed on each of the characteristics. Statistical significance was evaluated using Bonferroni adjustment at 0.05 divided by the total number of classes minus one [32].

**Table 1:** Beneficiary characteristics at death by place of care trajectory group

			Home			Skilled home care			Institutional care		
	Population	Sample	Class 1	Class 2	Class 3	Class 4	Class 5	Class 6	Class 7	Class 8	Class 9
Number (row %)	1,998,282 (100.0)	199,828 (100.0)	57,856 (29.0)	40,866 (20.5)	18,126 (9.1)	28,437 (14.2)	16,474 (8.2)	9544 (4.8)	9543 (4.8)	9827 (4.9)	9155 (4.6)
Table 1a.											
Median (IQR) days of skilled home care, last 3 years	39 (0-142)	38 (0-143)	0 (0–18)	14 (0–42)	113 (62–176)	133 (97–190)	371 (292–465)	756 (612–902)	56 (10–128)	30 (0-120)	0 (0–18)
Average days (SD) with skilled home care, last 3 years	117.8 (191.2)	117.7 (190.9)	12.0 (19.9)	24.0 (27.7)	130.3 (88.7)	147.2 (69.5)	382.1 (122.6)	453.7 (186.5)	82.5 (87.3)	81.0 (113.0)	23.6 (49.9)
Median (IQR) days of institutional care, last 3 years	31 (8-100)	31 (8-100)	6 (0–19)	29 (12–61)	52 (23–97)	33 (10–68)	65 (22–136)	50 (11–128)	269 (209–350)	606 (510–718)	728 (706–730)
Average days (SD) in institutional care, last 3 years	114.2 (198.0)	114.1 (197.9)	13.2 (17.0)	42.7 (40.3)	69.2 (63.8)	45.9 (44.2)	98.6 (106.2)	88.2 (102.7)	281.1 (89.3)	605.9 (121.5)	724.4 (91.4)
Care intensity (SE)^a^	115.4 (268.0)	115.3 (267.8)	38.4 (26.3)	109.4 (48.2)	268.7 (107.6)	239.0 (70.8)	579.3 (141.7)	631.0 (209.8)	644.7 (76.4)	1292.8 (151.9)	1472.4 (102.9)
Table 1b. - Number (column %)											
Age at death, mean (SD)	81.8 (10.2)	81.8 (10.2)	80.1 (9.9)	80.0 (10.3)*	83.0 (10.4)*	83.0 (9.4)*	84.1 (9.9)*	83.7 (10.8)*	82.3 (10.3)*	84.3 (10.1)*	85.0 (10.6)*
Age ≥ 70 (reference)	1,811,551 (90.7)	181,281 (90.7)	52,181 (90.2)	36,111 (88.4)	16,452 (90.8)	26,559 (93.4)	15,304 (92.9)	8604 (90.2)	8615 (90.3)	9044 (92.0)	8411 (91.9)
Age < 70	186,731 (9.3)	18,547 (9.3)	5675 (9.8)	4755 (11.6)*	1674 (9.2)	1878 (6.6)*	1170 (7.1)*	940 (9.8)	928 (9.7)	783 (8.0)*	744 (8.1)*
Sex											
Female (reference)	1,055,592 (52.8)	105,322 (52.7)	26,127 (45.2)	19,076 (46.7)*	10,471 (57.8)*	15,456 (54.4)*	10,027 (60.9)*	6120 (64.1)*	5277 (55.3)*	6419 (65.3)*	6349 (69.4)*
Male	942,690 (47.2)	94,506 (47.3)	31,729 (54.8)	21,790 (53.3)	7655 (42.2)	12,981 (45.6)	6447 (39.1)	3424 (35.9)	4266 (44.7)	3408 (34.7)	2806 (30.6)
Race/ethnicity											
White, non-Hispanic	1,620,601 (81.1)	162,016 (81.1)	46,253 (79.9)	33,307 (81.5)	14,668 (80.9)	23,671 (83.2)	13,675 (83.0)	7237 (75.8)	7803 (81.8)	8081 (82.2)	7321 (80.0)
Black, non-Hispanic	192,674 (9.6)	19,268 (9.6)	5062 (8.7)	3708 (9.1)	1866 (10.3)*	2470 (8.7)	1528 (9.3)	1295 (13.6)*	1081 (11.3)*	1107 (11.3)*	1151 (12.6)*
Hispanic	131,851 (6.6)	13,185 (6.6)	4702 (8.1)	2651 (6.5)*	1157 (6.4)*	1622 (5.7)*	911 (5.5)*	817 (8.6)	429 (4.5)*	419 (4.3)*	477 (5.2)*
Asian American/Pacific Islander	43,262 (2.2)	4366 (2.2)	1550 (2.7)	958 (2.3)*	349 (1.9)*	567 (2.0)*	274 (1.7)*	141 (1.5)*	188 (2.0)*	168 (1.7)*	171 (1.9)*
American Indian/Alaska Native	9894 (0.50)	993 (0.50)	289 (0.50)	242 (0.59)	86 (0.47)	107 (0.38)*	86 (0.52)	54 (0.57)	42 (0.44)	52 (0.53)	35 (0.38)
Medicare Insurance Type											
Medicare fee-for-service only (reference)	913,220 (45.7)	91,147 0 (45.6)	29,401 (50.8)	20,237 (49.5)	9084 (50.1)	14,527 (51.1)	8097 (49.2)	4152 (43.5)	3175 (33.3)	1536 (15.6)	938 (10.3)
Medicare FFS + Medicaid	366,672 (18.4)	36,691 (18.4)	4891 (8.5)	4812 (11.8)*	2688 (14.8)*	3284 (11.6)*	3127 (19.0)*	2813 (29.5)*	3458 (36.2)*	5612 (57.1)*	6006 (65.6)*
Medicare Advantage only	511,504 (25.6)	51,429 (25.7)	19,484 (33.7)	12,132 (29.7)*	4556 (25.2)*	8285 (29.1)*	3628 (22.0)*	1521 (15.9)*	1101 (11.5)*	443 (4.5)*	279 (3.0)*
Medicare Advantage + Medicaid	206,886 (10.3)	20,561 (10.3)	4080 (7.0)	3685 (9.0)*	1798 (9.9)*	2341 (8.2)*	1622 (9.8)*	1058 (11.1)*	1809 (19.0)*	2236 (22.8)*	1932 (21.1)*
Neighborhood Profile											
Metropolitan, ADI < 85% (reference)	1,381,642 (69.1)	138,546 (69.3)	39,743 (68.7)	28,049 (68.6)	13,305 (73.4)	20,226 (71.1)	11,856 (72.0)	6645 (69.6)	6454 (67.6)	6353 (64.6)	5915 (64.6)
Metropolitan, ADI > 85%	198,840 (10.0)	19,679 (9.9)	5813 (10.0)	4207 (10.3)	1585 (8.7)*	2760 (9.7)*	1546 (9.4)*	918 (9.6)	929 (9.7)	1027 (10.5)	894 (9.8)
Nonmetropolitan, ADI < 85%	251,827 (12.6)	25,042 (12.5)	7326 (12.7)	5169 (12.6)	1970 (10.9)*	3315 (11.6)*	1901 (11.5)*	1259 (13.2)	1275 (13.4)	1449 (14.7)*	1378 (15.1)*
Nonmetropolitan, ADI > 85%	119,005 (6.0)	11,871 (6.0)	3490 (6.0)	2462 (6.0)	861 (4.8)*	1474 (5.2)*	807 (4.9)*	551 (5.8)	667 (7.0)*	789 (8.0)*	770 (8.4)*
Chronic Conditions											
Total number of conditions, mean (SD)	5.1 (2.8)	5.1 (2.8)	3.7 (2.5)	5.0 (2.7)*	5.9 (2.6)*	5.1 (2.6)*	6.0 (2.5)*	6.5 (2.4)*	6.3 (2.5)*	6.5 (2.3)*	6.7 (2.2)*
Alzheimer’s disease and dementia	902,208 (45.2)	90,228 (45.2)	13,566 (23.4)	13,486 (33.0)*	9319 (51.4)*	13,623 (47.9)*	10,682 (64.8)*	6711 (70.3)*	6605 (69.2)*	8188 (83.3)*	8048 (87.9)*
Hypertension	1,645,124 (82.3)	164,333 (82.2)	41,198 (71.2)	33,511 (82.0)*	15,983 (88.2)*	23,579 (82.9)*	14,767 (89.6)*	8858 (92.8)*	8683 (91.0)*	9139 (93.0)*	8615 (94.1)*
Hyperlipidemia	1,479,637 (74.0)	147,796 (74.0)	36,736 (63.5)	30,443 (74.5)*	14,573 (80.4)*	21,299 (74.9)*	13,327 (80.9)*	8068 (84.5)*	7802 (81.8)*	8104 (82.5)*	7444 (81.3)*
Chronic kidney disease	1,086,192 (54.4)	108,566 (54.3)	22,821 (39.4)	22,937 (56.1)*	11,544 (63.7)*	15,401 (54.2)*	10,222 (62.0)*	6471 (67.8)*	6520 (68.3)*	6519 (66.3)*	6121 (66.9)*
Congestive heart failure	1,024,014 (51.2)	102,499 (51.3)	19,337 (33.4)	30,981 (75.8)*	11,510 (63.5)*	14,674 (51.6)*	10,479 (63.6)*	6646 (69.6)*	6294 (66.0)*	6434 (65.5)*	6143 (67.1)*
Depression	921,497 (46.1)	91,932 (46.0)	16,044 (27.7)	16,337 (40.0)*	9610 (53.0)*	12,955 (45.6)*	9802 (59.5)*	6527 (68.4)*	6076 (63.7)*	7267 (73.9)*	7314 (79.9)*
Diabetes	904,000 (45.2)	90,347 (45.2)	19,511 (33.7)	18,250 (44.7)*	9576 (52.8)*	12,399 (43.6)*	8489 (51.5)*	5627 (59.0)*	5395 (56.5)*	5609 (57.1)*	5491 (60.0)*
Chronic obstructive pulmonary disease	848,079 (42.4)	84,508 (42.3)	17,107 (29.6)	17,816 (43.6)*	9199 (50.8)*	12,100 (42.6)*	8434 (51.2)*	5538 (58.0)*	4828 (50.6)*	4931 (50.2)*	4555 (49.8)*
Stroke/TIA	552,646 (27.7)	55,529 (27.8)	9785 (16.9)	10,247 (25.1)*	6228 (34.4)*	7827 (27.5)*	5935 (36.0)*	3819 (40.0)*	3576 (37.5)*	4033 (41.0)*	4079 (44.6)*
Cancer	448,861 (22.5)	44,729 (22.4)	11,863 (20.5)	9742 (23.8)*	4307 (23.8)*	7323 (25.8)*	3939 (23.9)*	2091 (21.9)*	2021 (21.2)	1881 (19.1)*	1558 (17.0)*
Acute myocardial infarction	236,073 (11.8)	23,619 (11.8)	4494 (7.8)	5403 (13.2)*	2805 (15.5)*	3315 (11.7)*	2357 (14.3)*	1446 (15.2)*	1449 (15.2)*	1240 (12.6)*	1110 (12.1)*
End-stage renal disease	68,147 (3.4)	6896 (3.5)	750 (1.3)	1878 (4.6)*	1053 (5.8)*	840 (3.0)*	582 (3.5)*	400 (4.2)*	722 (7.6)*	414 (4.2)*	257 (2.8)*
HIV	55,207 (2.8)	5575 (2.8)	1190 (2.0)	1361 (3.3)*	636 (3.5)*	648 (2.3)	455 (2.8)*	290 (3.0)*	398 (4.2)*	312 (3.2)*	285 (3.1)*

^a^ Care intensity is calculated as the weighted average of days with skilled home care (weighted by a factor of one) and in institutional/inpatient care (weighted by a factor of two). These weights are arbitrary and are assigned for the sake of illustration

* Statistically significant differences between each class and the reference class in bivariate multinomial logistic regression., home class 1, using a Bonferroni-corrected alpha of 0.00625 (= 0.05/8) as there were nine classes.^32^

The Bolck-Croon-Hagenaars (BCH) method [33, 34] was recently developed to correct for bias in assessing the statistical association between class membership and exogenous covariates due to sample variance or misclassification in estimating class memberships. However, the BCH method was not developed for bivariate outcomes as used in this study [35].

## Results

### Study population and characteristics

Our analytic cohort consisted of 199,828 Medicare beneficiaries who died in 2018. Nearly half of the cohort had fee-for-service Medicare only [45.6%], 18.4% had fee-for-service Medicare and Medicaid, 25.7% had Medicare Advantage only, and 10.3% had Medicare Advantage and Medicaid. Their mean (standard deviation, SD) age was 81.8 (10.2) years, and 105,322 [52.7%] were female. The cohort consisted of predominantly non-Hispanic white [81.1%] individuals, followed by non-Hispanic Black [9.6%], Hispanic [6.6%], Asian American/Pacific Islander [2.2%], and American Indian/Alaska Native [0.5%] individuals. The majority of beneficiaries in the cohort lived in socioeconomically advantaged metropolitan [69.1%] and nonmetropolitan areas [12.6%] and ranked below the 85th percentile nationally on the Area Deprivation Index (ADI 3.0) [30]. The remainder lived in metropolitan [10%] and nonmetropolitan [6%] areas ranked at or above the 85th percentile nationally on the ADI 3.0. The remaining 2.3% of the cohort lived in areas where the ADI 3.0 ranking was suppressed for privacy reasons [30]. On average (SD), beneficiaries in the cohort had 5.1 [2.8] chronic conditions. The most prevalent chronic conditions were hypertension [82.3%] and hyperlipidemia [74.0%], followed by chronic kidney disease [54.3%], congestive heart failure [51.3%], ADOD [45.2%], diabetes [45.2%], chronic obstructive pulmonary disease [42.4%], history of acute myocardial infarction [27.7%], and history of cancer [22.2%]. The first columns of Table 1 include more details about the analytic cohort and population cohort of Medicare decedents.

### Place of care trajectories

All three random samples resulted in similar trajectory classes, with two of the samples producing nearly identical results and a maximum of nine classes. The third sample produced similar results, with an empty class appearing when a ten-class model was fit. Relative entropy was highest for the nine-class solution [35, 36]. The BIC for the six to nine class models was respectively 6,692,658; 6,694,291; 6,629,166; and 6,377,736 with the nine class model having the lowest BIC. Relative entropy was calculated using the posterior probabilities adjusted by sample size and measured how well the classes separated from each other.

**Fig. 2:**
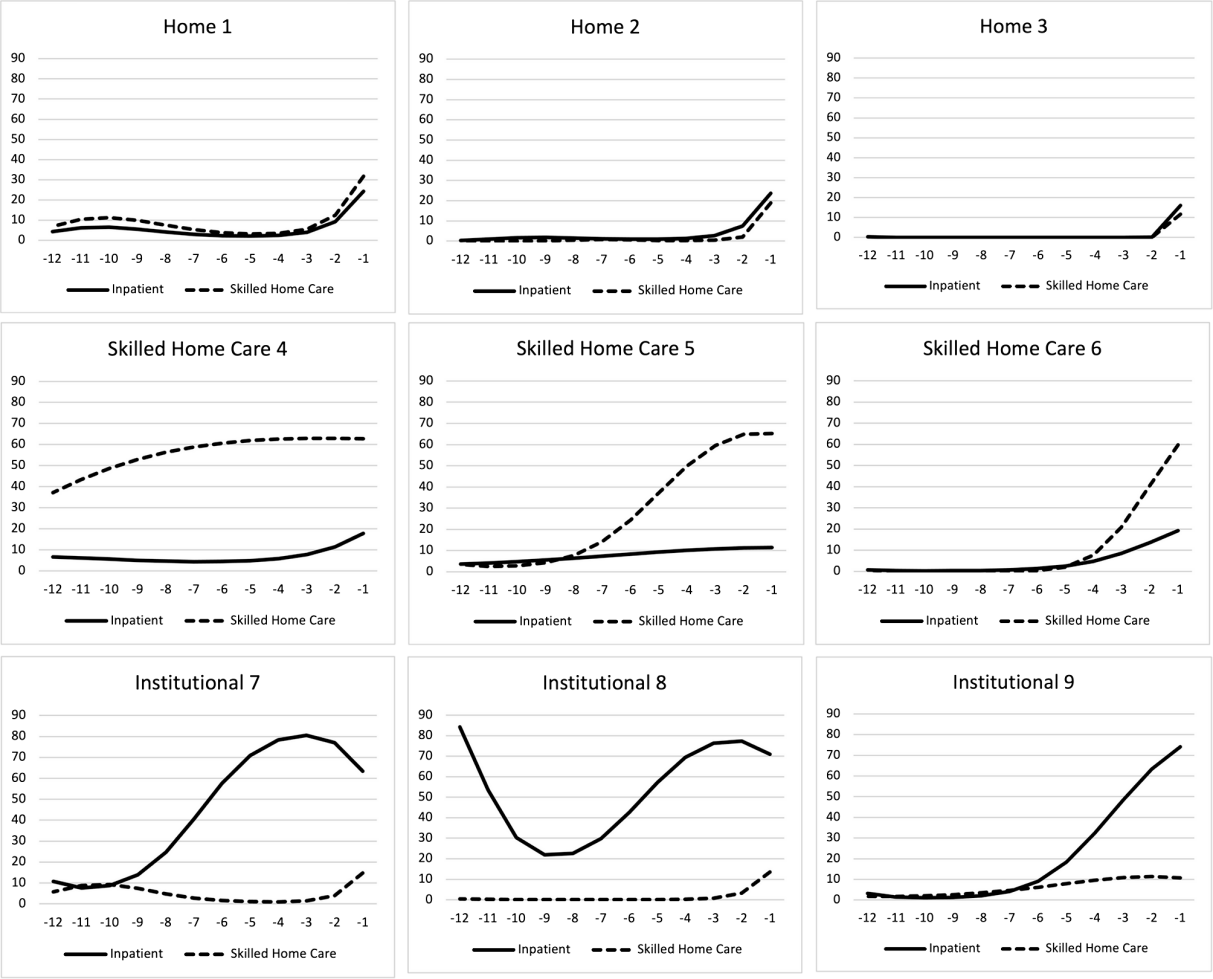
Place of care trajectories by latent class group. Y-axis represents the days in skilled homecare or institutional care in a given quarter before death (30 days would fall at the 33% line, and 45 days at the 50% line). X-axis represents the quarter (3-month period) before death, going back 3 years in time from date of death. See additional details about the nine classes in Table 1a

The final nine classes (see Fig. 2) were numerically labeled and arranged conceptually into three major clusters: home, skilled home care, and institutional care. The proportion of beneficiaries in each of the nine classes is described in Table 1a. The horizontal axis represents the quarter (3-month period) before death and the vertical axis represents the days in skilled homecare or institutional care in a given quarter before death. The three classes in the “home” cluster were distinguished by care mostly at home with skilled home care and/or institutional care concentrated in the last quarter of life. The three classes in the “skilled home care” cluster were characterized by the extended use of home health care and/or home hospice, and the three classes in the “institutional” cluster were characterized by the extended use of institutional or inpatient care.

As expected, there were large differences in the mix and types of health services used across classes, with thirty-seven-fold variation in the average use of skilled home care ranging from 12 days in class 1 of the home cluster to 454 days in class 6 of the skilled home care cluster and fifty-five-fold differences in the average use of institutional care ranging from 13 days in class 1 to 724 days in class 9 of the institutional cluster during the last three years of life. Care intensity for each class, which we report in Table 1a, increased as the class label increased numerically; however, class 3 had higher care intensity with higher total usage of both institutional care and skilled home care than class 4. The first home class, class 1 (57,856 [29.0%]), had the lowest average use of skilled home care (12 days) and institutional care (13 days) during the last three years of life, all of which occurred in the last four months of life. The second home class, class 2 (40,866 [20.5%]), used an average of 24 days of skilled home care and 40 days of institutional care, mostly during the last year of life. The final home class, class 3 (18,126 [9.1%]), used an average of 130 days of skilled home care and 69 days of institutional care, mostly during the last year of life, with low-level usage during the third year before death.

Within the skilled home care cluster, class 4 (28,437 [14.2%]) had the lowest average use of skilled home care (147 days) and institutional care (46 days), mostly during the last year of life. The second skilled home care class, class 5 (16,474 [8.2%]), used an average of 382 days of skilled home care and 106 days of institutional care, mostly during the last two years of life. The final skilled home care class, class 6 (9544 [4.8%]), used an average of 454 days of skilled home care and 103 days of institutional care almost consistently during the last three years of life. Across the three skilled home care classes, the use of institutional care during the last quarter of life was similar, ranging from 13 to 19 days on average.

Within the institutional cluster, class 7 (9543 [4.8%]) used an average of 83 days of skilled home care and 281 days of institutional care, mostly during the last one and a half years of life. The second institutional class, class 8 (9827 [4.9%]), used an average of 81 days of skilled home care and 606 days of institutional care, mostly during the last two years of life. The final institutional class, class 9 (9155 [4.6%]), used an average of 24 days of skilled home care and 724 days of institutional care throughout the last three years of life. Across the institutional classes, the use of skilled home care (including home hospice provided in a skilled nursing/nursing home facility) during the last 3 months of life was similar, ranging from 11 to 17 days on average.

Beneficiaries with ADOD (n = 89,923) appeared in greater numbers within the first five classes, as these classes had large sizes; however, they made up a larger proportion of the skilled home care classes (4–6) [57%] and the institutional classes (7–9) [80%] compared to the home classes [31%] shown in Table 1b and the last panel of the Additional Fig. 1 [see Additional file 2].

### Correlates of place of care trajectory class membership

Trajectory classes with greater care intensity (classes 5–9) tended to have a greater proportion of beneficiaries who were female, of Black race, Medicaid-dual eligible, and living with multiple chronic conditions, especially ADOD. Among Medicare beneficiaries with dementia, the row percentage in Table 1b indicates that 41% had relatively low health care utilization until the last quarter of life (home classes). One-third (34%) received extended home health care and/or home hospice services (skilled home care classes), and one-quarter (26%) received extended care in a nursing home or inpatient setting (institutional classes).

The cross-tabulation in Table 1 shows that the cluster of home without skilled care classes had the lowest proportion of Medicaid dual eligible beneficiaries compared to the skilled home care and institutional care clusters. Home class 1 had highest percentage of beneficiaries with male sex, Hispanic or Asian American/Pacific Islander (AAPI) ethnicity, and Medicare Advantage insurance; and lowest number of chronic conditions among all classes. Home class 2 had the youngest average age at death, second highest percentage of male sex, and second highest percentage of Medicare Advantage. Home class 3 had the smallest percentage of beneficiaries living in disadvantaged neighborhoods (ADI percentile ≥ 85%) and the highest percentage living in metropolitan areas among all the classes.

The proportion of female sex was lowest in the home cluster, higher in the skilled home care cluster, and highest in the institutional cluster. Skilled home care class 4 had highest percentage of white race among all the classes and highest percentage of Medicare Advantage among the classes in the home cluster and the institutional cluster. Skilled home care class 5 had second highest percentage of white race among all the classes, and second highest percentage of Medicare Advantage among the classes in the home cluster and the institutional cluster. Skilled home care class 6 had the highest percentage of Black race and Hispanic ethnicity among all the classes. Beneficiaries in both class 5 and class 6 had six or more chronic conditions on average.

Overall, the institutional cluster had the highest percentage of beneficiaries with female sex, Black race, Medicaid dual eligibility, residence in a nonmetropolitan area, residence in a disadvantaged area, and highest number of chronic conditions compared to the other two clusters (home, with or without skilled care). For ease of interpretation, the results described in this section and Table 1 are also plotted as bar charts in the Additional Figure [see Additional File 2].

### Distribution of place of care trajectory class membership by state

**Fig. 3:**
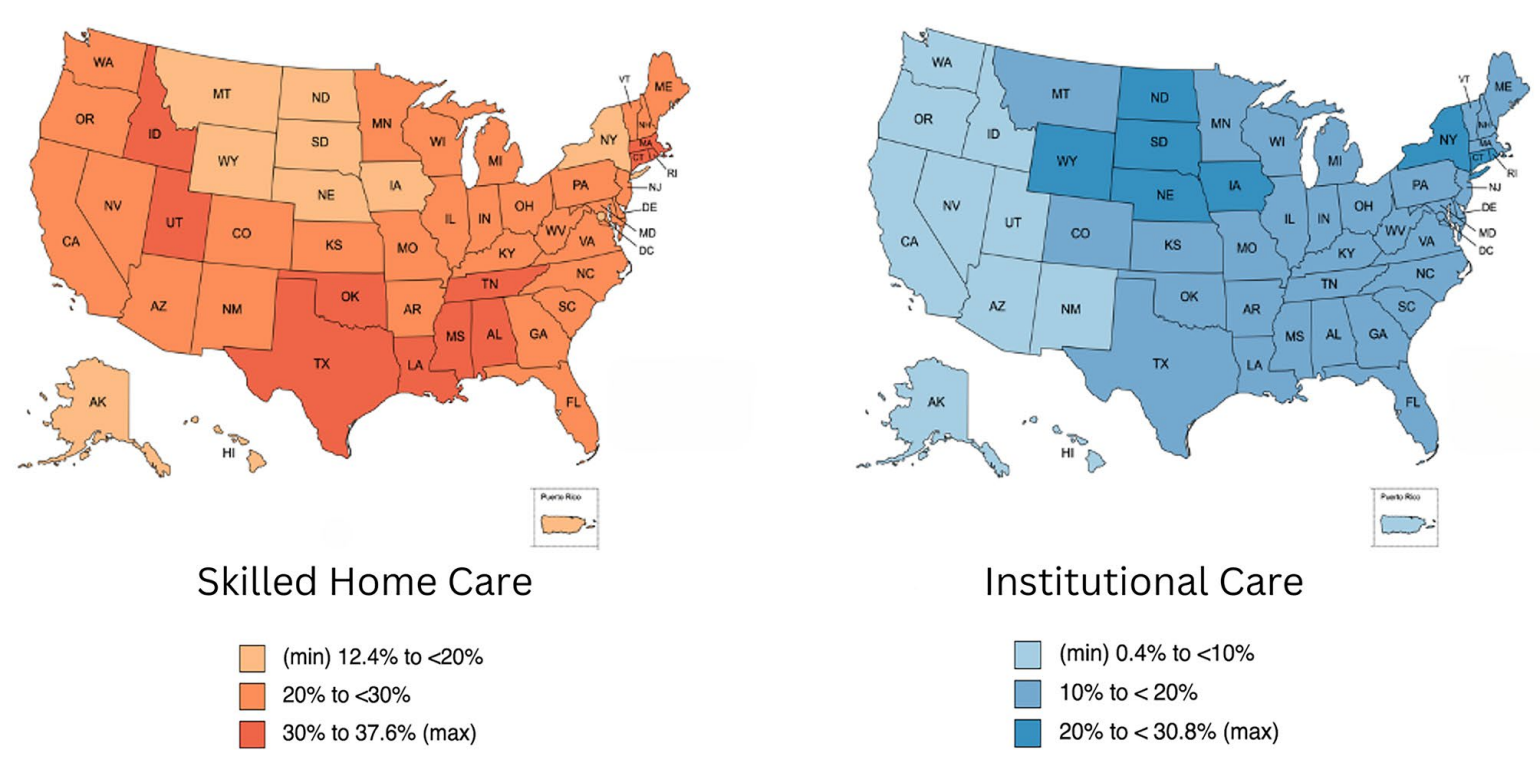
Place of care trajectory patterns by state

Nationally, over half (59%) of Medicare beneficiaries were in the home cluster, one-quarter (27%) were in the skilled home care cluster, and the rest (14%) were in the institutional cluster. There were large variations by state in the use of services during the last three years of life. By state, home classes were most frequent among beneficiaries from Alaska (81.5%), Puerto Rico (81.4%), Hawaii (72.9%), Arizona (69.2%), and Oregon (68.9%) and least frequent among beneficiaries from Massachusetts (47.1%), Louisiana (47.8%), Rhode Island (48.3%), and Connecticut (48.6%). Skilled home care classes were most frequent among beneficiaries from the southern states of Oklahoma (39.9%), Louisiana (37.2%), Texas (37.7%), Alabama (36.8%), and Mississippi (36.4%) and least frequent among beneficiaries from North Dakota (8.3%), South Dakota (12.1%), Alaska (12.4%), Wyoming (13.0%), and Hawaii (17.6%). Institutional classes were most frequently observed among beneficiaries in the midwestern states of North Dakota (30.8%), South Dakota (30.0%), Iowa (25.1%), and Nebraska (22.3%), and New York (21.6%) and least frequent among beneficiaries from Puerto Rico (0.5%), Arizona (5.2%), Oregon (5.4%), Alaska (6.0%), and Nevada (6.4%). The full results are summarized in Fig. 3 and displayed in the Additional Table 1 [see Additional File 1].

## Discussion

The idea of describing patterns of trajectories in relation to death rather than age or diagnosis originated with Glaser and Strauss’s seminal book *Time for Dying* (1968) [37]; however, few studies have applied this approach to analyzing aging trajectories. Two laudable examples include Lunney and colleagues’ multisite Established Populations for Epidemiologic Studies of the Elderly study (2003) [38] and Gill et al. (2010) [14], who described disability trajectories during the last year of life among decedents from the Precipitating Events Project. Understanding where and when patients receive care in institutional versus home settings has important implications for care planning, costs of care, and quality of life. The present study fills a gap in the literature by characterizing Medicare beneficiaries’ place of care trajectories for the last three years of life.

Using GBTM to examine place of care trajectories during the last three years of life, we found nine distinct care trajectories that varied in the timing and duration of home health care/home hospice and institutional/inpatient care. To our knowledge, this is the first study to use a large, nationally representative cohort to characterize Medicare beneficiaries’ late life place of care trajectories and associated sociodemographic and clinical factors. Trajectory classes with more intensive care tend to have higher proportion of beneficiaries being female, Black race, dual eligible, living in non-metro areas, and increasingly higher prevalence of clinical/chronic conditions especially dementia. The shortage of health care providers in rural areas may explain the observation that non-metro areas had a higher percentage of beneficiaries in the institutional care setting (e.g., nursing home) trajectories.

Overall, we found that over half (59%) of beneficiaries spent their last 3 years mostly at home, while one-quarter (27%) used skilled home care and one in seven (14%) had institutional or inpatient care mostly throughout the last three years of life. Our findings are similar to those reported in a recent prospective cohort study using a representative sample from the National Health and Aging Trends Study (NHATS), which also found that 58% of NHATS participants remained at home and 17% transitioned to or died in an institutional setting [39]. Our findings are also consistent with the recent downward trend of deaths in acute care hospitals and upward trend of deaths in home and community settings [40]. Evidence has shown both advantages and disadvantages of either institutional or home care [41–44]. Hospitals are subject to quality measurement programs that may create indirect incentives for home death [45].

Furthermore, while we found that beneficiaries with Alzheimer’s disease and other dementias (ADOD) made up a large fraction (60%) of the skilled home care and institutional classes; 40% of beneficiaries with ADOD used minimal amounts of skilled home care or institutional care prior to the last few months of life, with 34% using extended periods of skilled home care, and 25% using extended periods of inpatient care. Advance care planning may offer opportunities to educate older adults about care options, including palliative and hospice care, and ensure that vulnerable patients with complex care needs and limited social support receive goal-concordant care. Prior studies have noted the failures and challenges in engaging hospitalized older adults in advance care planning [46]. Our findings emphasize the need for a structured and comprehensive approach to care discussions to identify and document patient care preferences and goals in all care settings, which can also be integrated into Medicare Annual Wellness Visits (AWVs) [47, 48], considering patient and family interest and eligibility for long-term support and services available through local, state and federal policy initiatives [49]. This is especially important for patients with dementia, who are more frequently hospitalized, rehospitalized, and discharged to a long-term care facility than patients without cognitive impairment [50, 51].

We found large variations between states in place of care trajectories (Fig. 3, Additional Table) during the last three years of life, highlighting the states (e.g., Alaska, Arizona, and Puerto Rico) that may have excessive barriers for patients and families seeking placement in a nursing home or long-term institutional care facility [52–54]. Additionally, some states (e.g., Alaska, North Dakota, and South Dakota) may have excessive barriers to accessing home health care and home hospice, which may be related to shortages of nursing staff, including registered nurses, home health aides, and home hospice aides [55–57]. The findings for New York state are consistent with recent efforts by the state to reduce overreliance on nursing home care, i.e., the Nursing Home Transition and Diversion (NHTD) Medicaid Waiver Program.

### Limitations

Several limitations warrant mention. First, the place of care trajectories were summarized for analysis in this study at a quarterly interval that did not focus on care transitions [10, 36]. Second, a methodological limitation of the latent class approach is that the number of identified classes may reflect floor or ceiling effects in available data [58, 59]. However, this limitation was minimized in our study due to the large sample size and replication of results across multiple random samples. Third, regarding our analysis of factors associated with the identified place of care trajectory, we would have liked to have information such as family and social support and the patient’s care preferences and their documentation. However, such information was not ascertainable with Medicare administrative data. Also, Medicare Advantage data may not capture all encounter with healthcare providers. Fourth, our care intensity variable was a novel attempt, but further arbitrary. Finally, we studied a five-year period prior to the coronavirus disease 2019 (COVID-19) pandemic, so the generalizability of our findings to the pandemic when the use of skilled home care and institutional services was greatly disrupted is unknown.

## Conclusions

Our analysis identified three major place of care trajectory patterns during the last three years of life among deceased Medicare beneficiaries. While the majority of older adults spent their final years at home with minimal use of skilled home care or institutional care until the final months of life, 40% had major health service needs. Extended use of skilled home care or institutional care was more frequent among older adults living with multiple chronic conditions, including dementia. A better understanding of the health care systems and policy factors that influence place of care trajectories may help to advance the triple aim [60] of improving the experience of care and health of the population and reducing the costs of care.

### Electronic supplementary material

Below is the link to the electronic supplementary material.

Supplementary Material 1

Supplementary Material 2

Supplementary Material 3

## Data Availability

The data that support the findings of this study are available from the Centers for Medicare and Medicaid Services, but restrictions apply to the availability of these data, which were used under license for the current study and are not publicly available. Data are, however, available from the authors upon reasonable request and with permission from the Centers for Medicare and Medicaid Services.

## Abbreviations

ADOD: Alzheimer’s disease and other dementias
CMS: Centers for Medicare and Medicaid Services
GBTM: Group-based trajectory modeling
MBSF: Medicare Beneficiary Summary File
EDB: Enrollment Database
MDS: Minimum Data Set
MDS-SB: Minimum Data Set – Swing Bed
OASIS: Outcome and Assessment Information Set
IRF-PAI: Inpatient Rehabilitation Facility Patient Assessment Instrument
MedPAR: Medicare Provider Analysis and Review file
