# Supplementary material for: Place of care in the last three years of life for Medicare beneficiaries
Source: BMC Geriatr. 2024 Jan 25;24:91. doi: 10.1186/s12877-023-04610-w (PMC10809551; doi:10.1186/s12877-023-04610-w)
Supplement: Supplementary file 1 — Supplementary Material 1 [file 12877_2023_4610_MOESM1_ESM.docx]

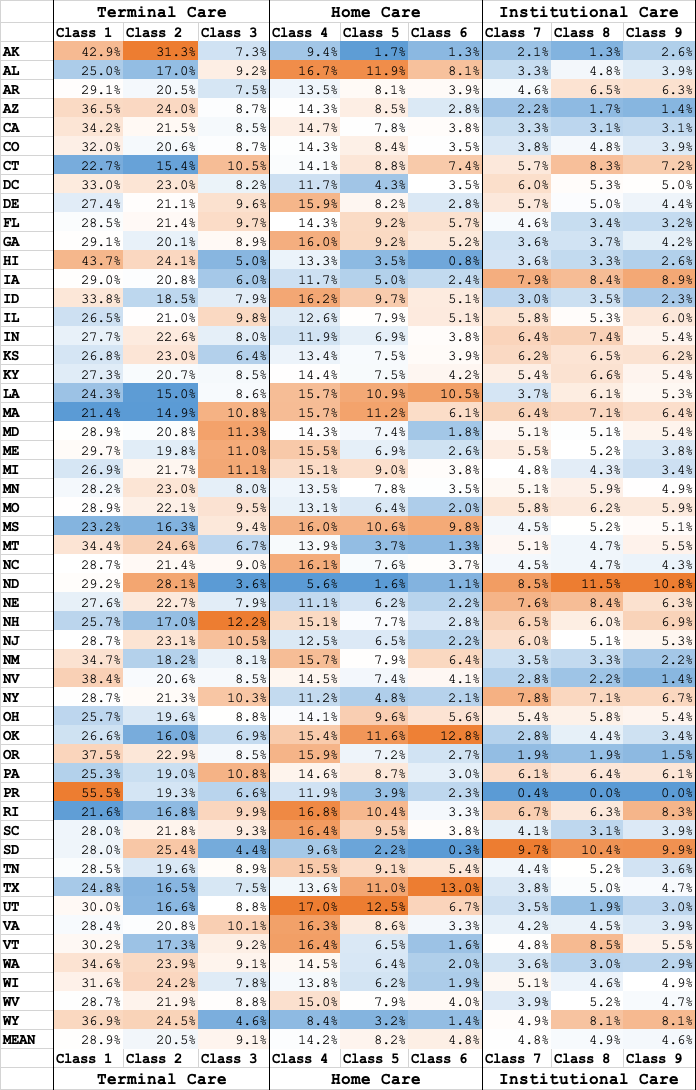
Additional Table 1. Within-state variation in the proportions of decedents belonging to each place of care trajectory class compared to the national average, color coded for each class

|  | **Home** | **Skilled home care** | **Institutional** |
| --- | --- | --- | --- |
