# Supplementary material for: Place of care in the last three years of life for Medicare beneficiaries
Source: BMC Geriatr. 2024 Jan 25;24:91. doi: 10.1186/s12877-023-04610-w (PMC10809551; doi:10.1186/s12877-023-04610-w)

Additional Figure 1. Plots of patient characteristics by care setting trajectory group (class)

Sex Age


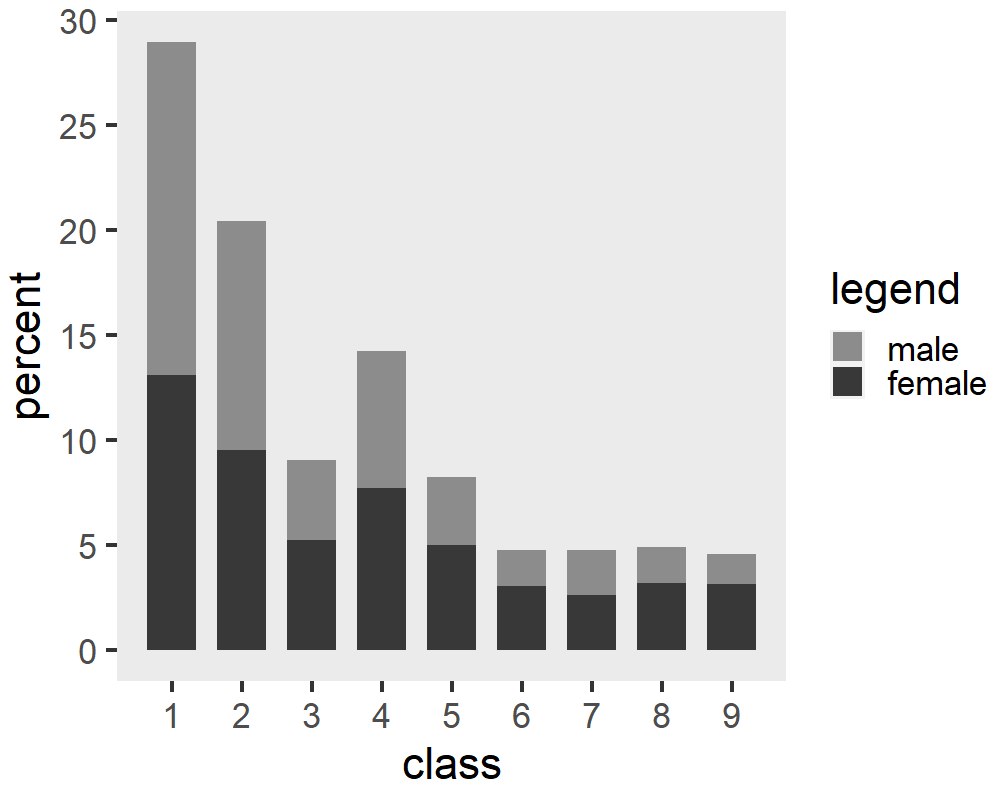

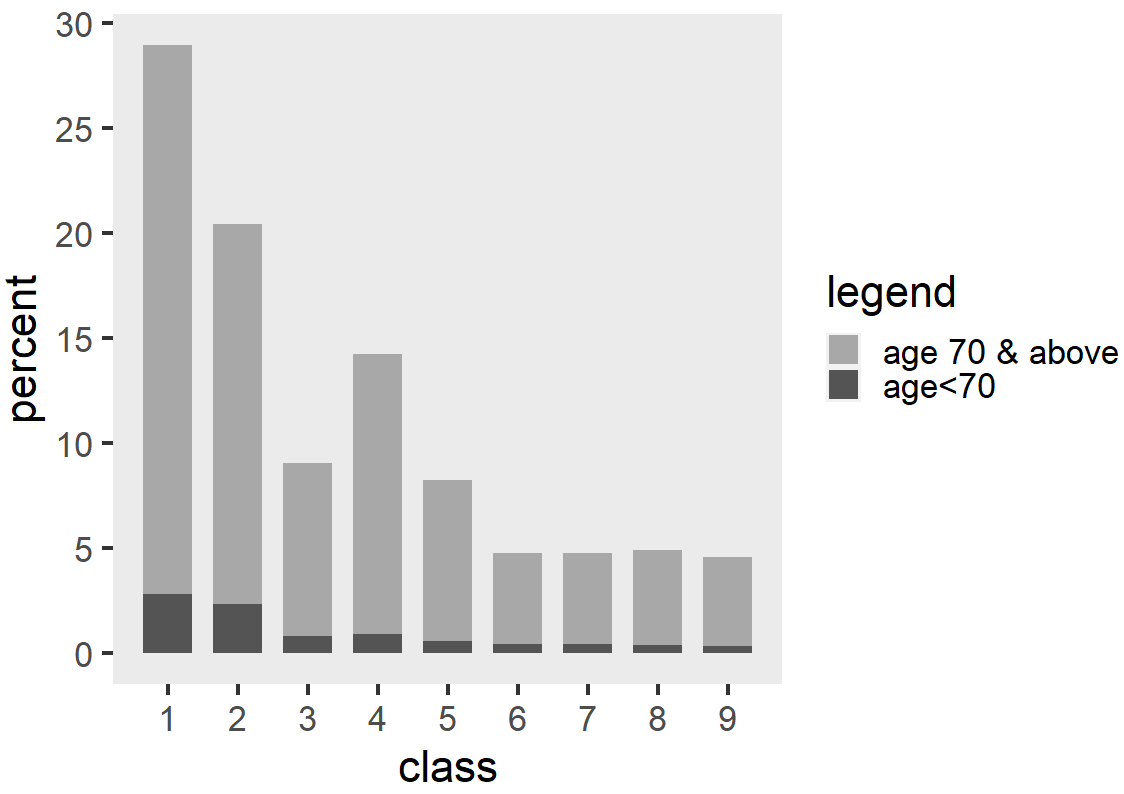


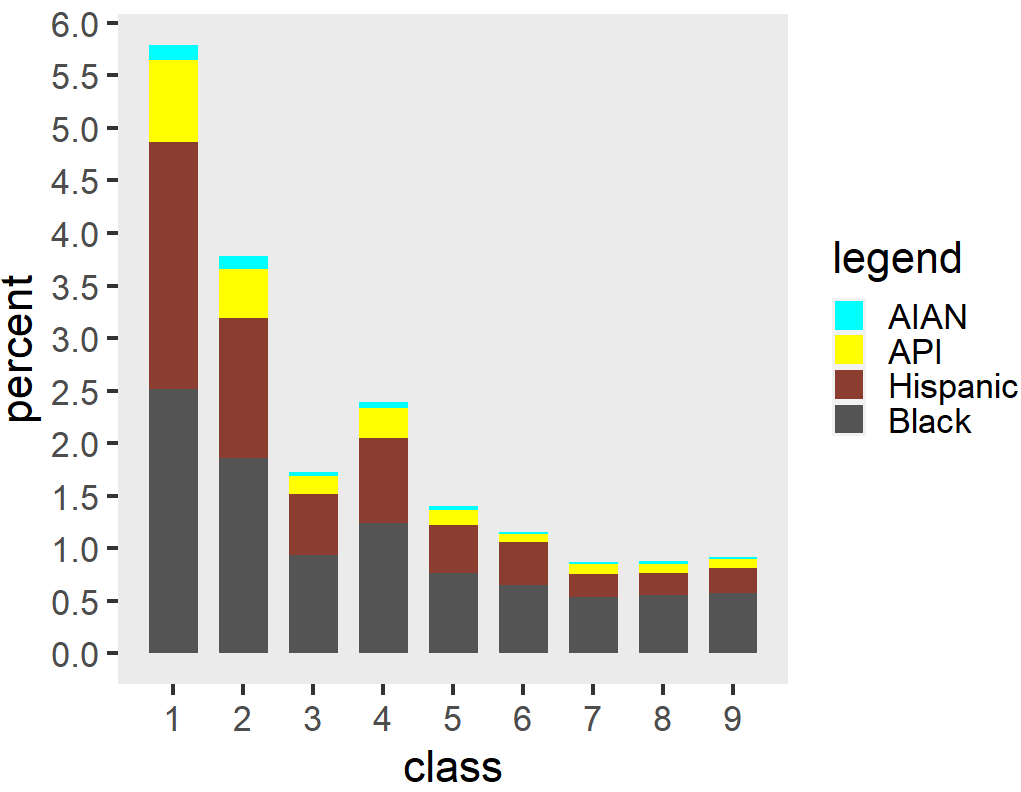

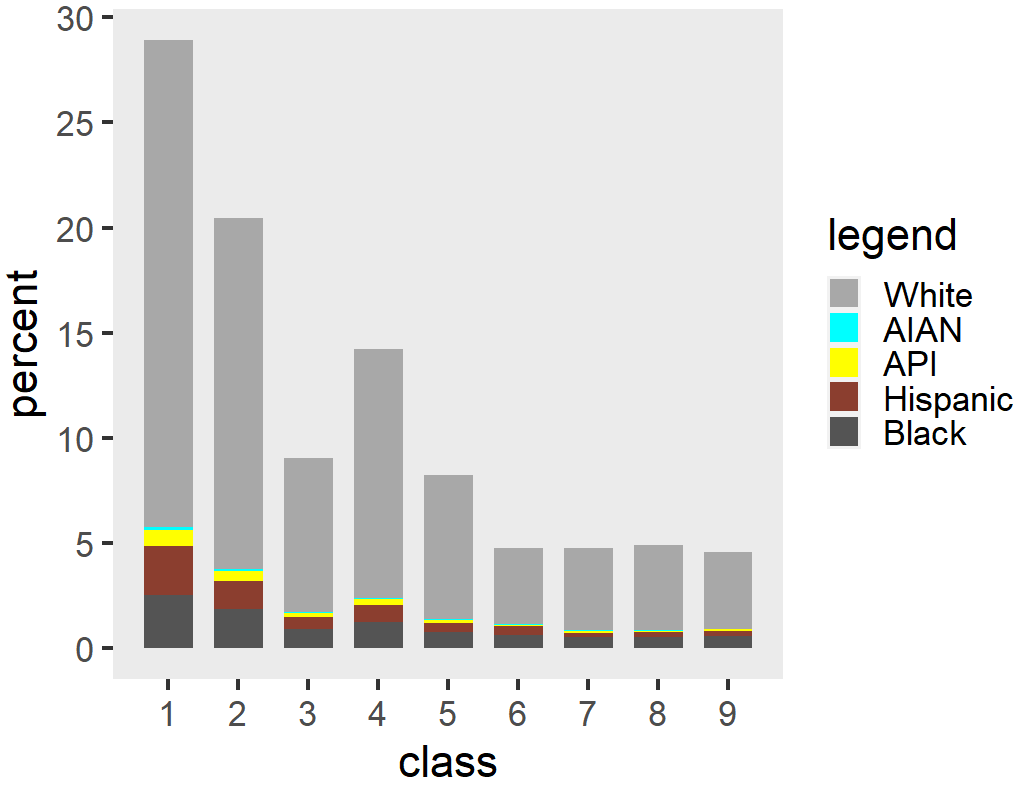
 All Races Racial Minorities

Insurance Type Neighborhood Profile


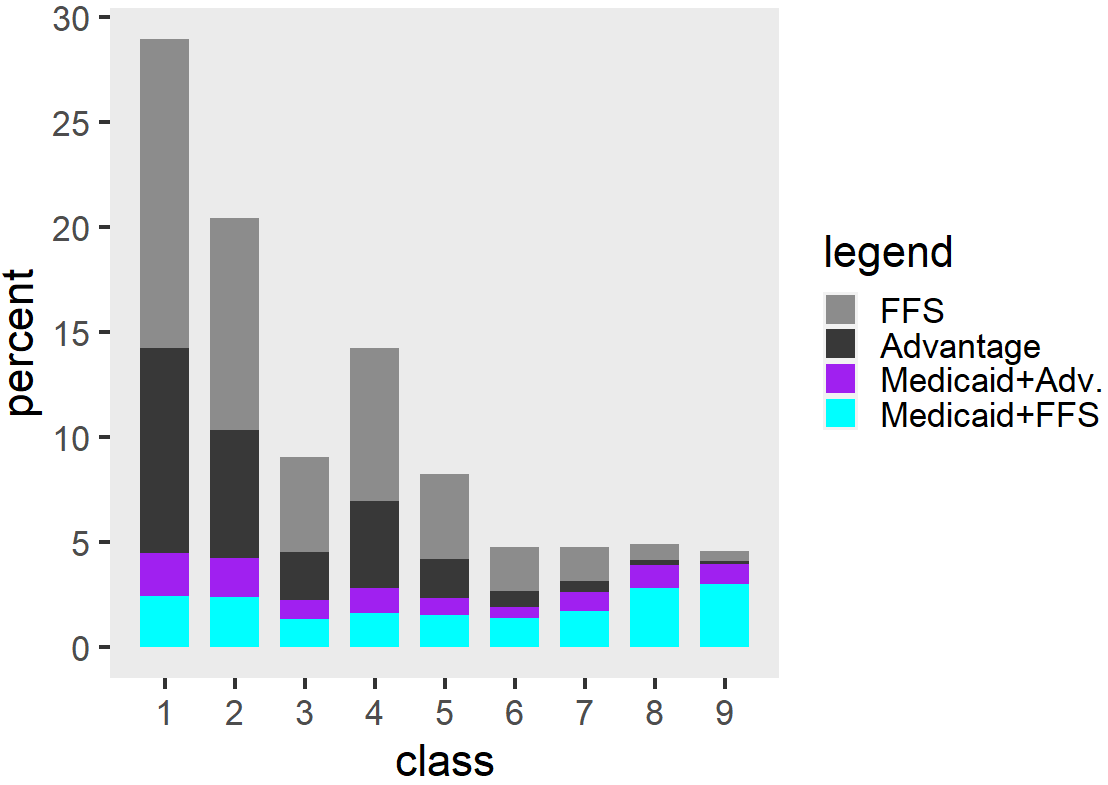

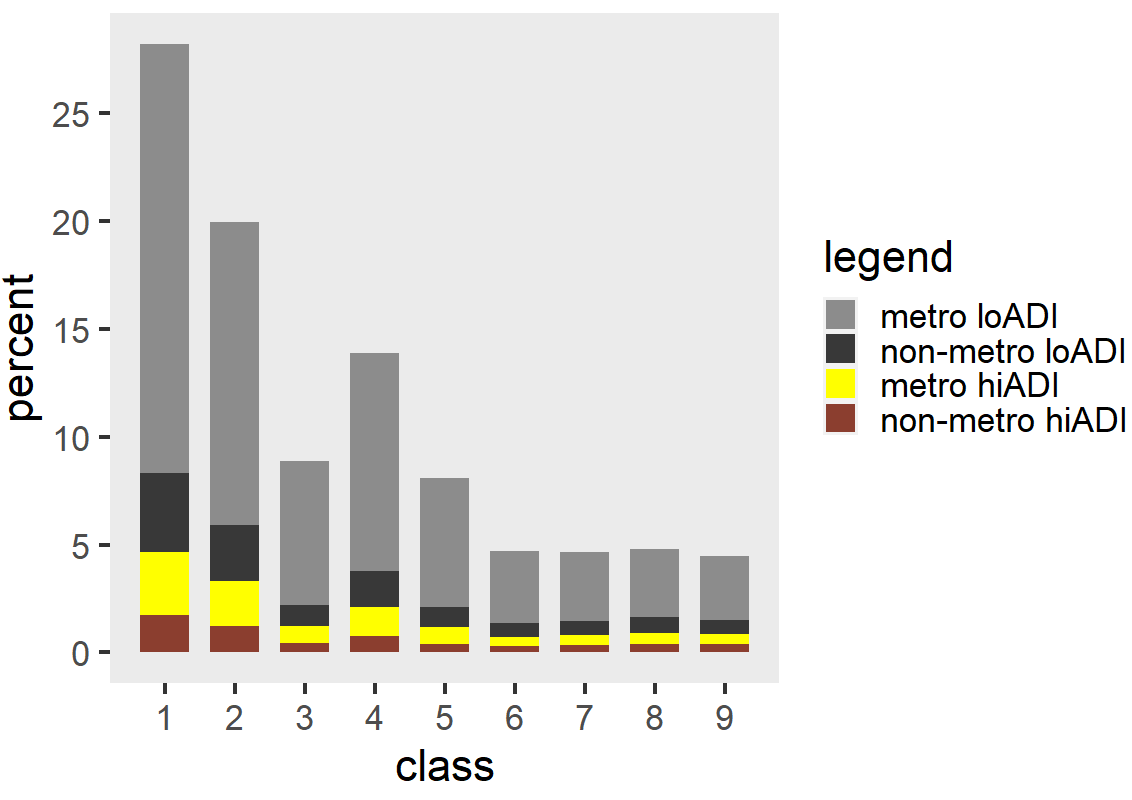


Number of Conditions Alzheimer’s disease and dementia


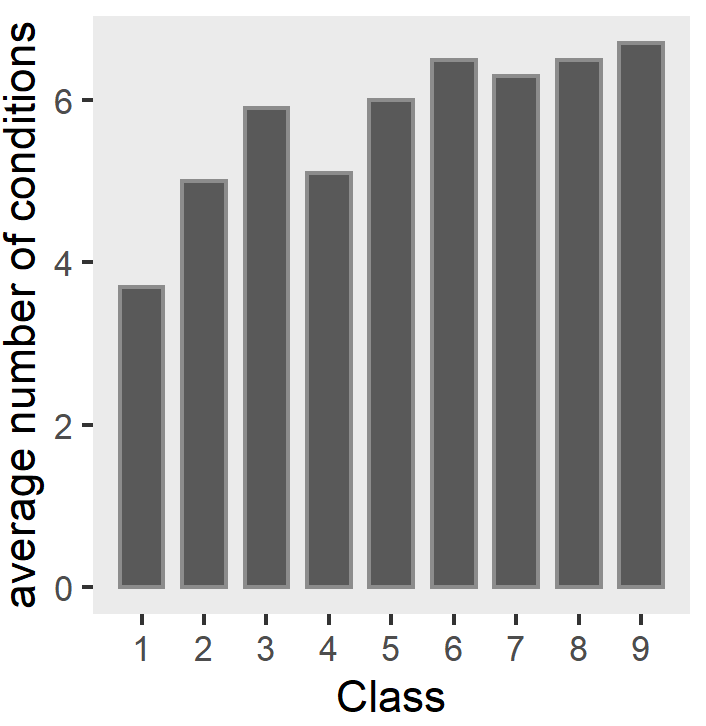

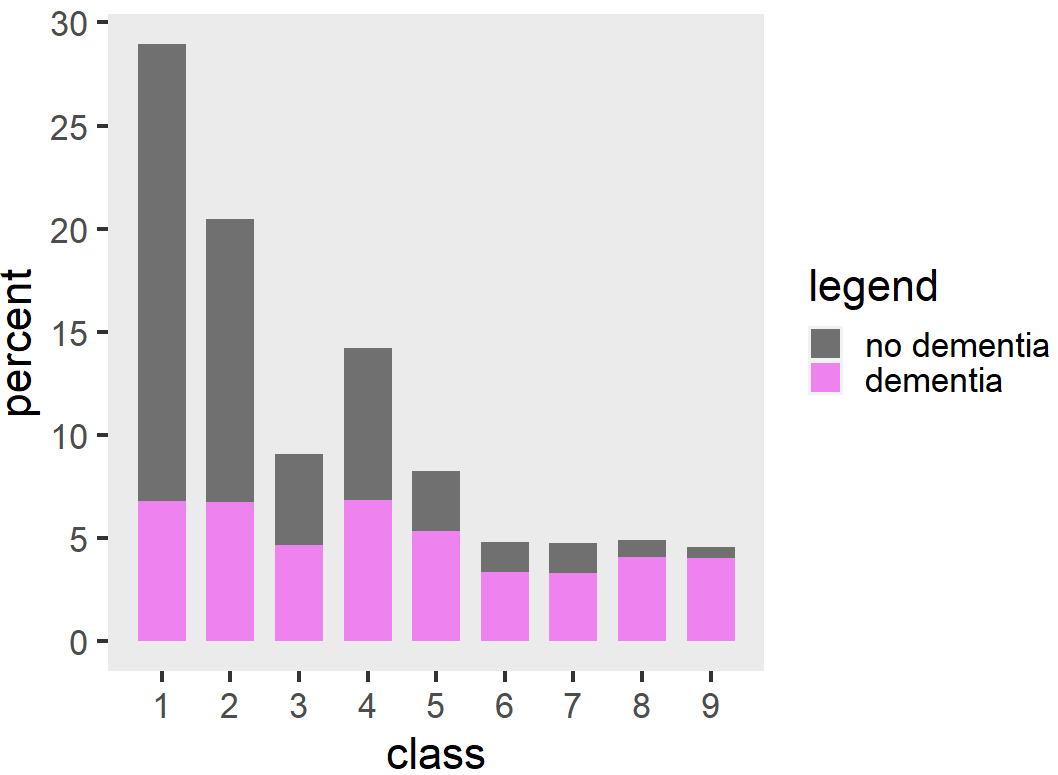

Supplement: Supplementary file 2 — Supplementary Material 2 [file 12877_2023_4610_MOESM2_ESM.docx]
