# Supplementary material for: Place of care in the last three years of life for Medicare beneficiaries
Source: BMC Geriatr. 2024 Jan 25;24:91. doi: 10.1186/s12877-023-04610-w (PMC10809551; doi:10.1186/s12877-023-04610-w)
Supplement: Supplementary file 3 — Supplementary Material 3 [file 12877_2023_4610_MOESM3_ESM.docx]

**Identification of Medicare Beneficiaries with Alzheimer’s Disease and Other Dementias**

The Medicare Beneficiary Summary File (MBSF) Chronic Conditions Warehouse flag for Alzheimer’s disease and related dementias (ALZH_DEMEN_FL) was augmented with additional dementia diagnoses present in MedPAR, MDS, and OASIS datasets using the International Classification of Diseases 10^th^ revision (ICD-10) codes 331.0, 331.11, 331.19, 331.7, 331.2, 290.0, 290.11, 290.13, 290.20, 290.21, 290.3, 290.40, 290.41, 290.42, 290.43, 294.0, 294.10, 290.11, 294.20, 294.21, 294.8, 797, G30.0, G30.1, G30.8, G30.9, F01.50, F01.51, F02.80, F02.81, F03.90, F03.91, F04, G13.8, F05, F06.1, F06.8, G30.0, G30.1, G30.8, G30.9, G31.1, G31.2, G31.01,G31.09, G94, R41.81, and R54.
